# Synergistic Antimicrobial Activity of *Camellia sinensis* and *Juglans regia* against Multidrug-Resistant Bacteria

**DOI:** 10.1371/journal.pone.0118431

**Published:** 2015-02-26

**Authors:** Amber Farooqui, Adnan Khan, Ilaria Borghetto, Shahana U. Kazmi, Salvatore Rubino, Bianca Paglietti

**Affiliations:** 1 Immunology and Infectious Diseases Research Laboratory, Department of Microbiology, University of Karachi, Karachi, Pakistan; 2 Department of Biomedical Sciences, University of Sassari, Sassari, Italy; 3 Division of Immunology, International Institute of Infection and Immunity, Shantou University Medical College, Shantou, Guangdong, China; 4 Department of Infection and Immunity, King Faisal Specialist Hospital and Research Center, Riyadh, Saudi Arabia; Institut National de la Recherche Agronomique, FRANCE

## Abstract

Synergistic combinations of antimicrobial agents with different mechanisms of action have been introduced as more successful strategies to combat infections involving multidrug resistant (MDR) bacteria. In this study, we investigated synergistic antimicrobial activity of *Camellia sinensis* and *Juglans regia* which are commonly used plants with different antimicrobial agents. Antimicrobial susceptibility of 350 Gram-positive and Gram-negative strains belonging to 10 different bacterial species, was tested against *Camellia sinensis* and *Juglans regia* extracts. Minimum inhibitory concentrations (MICs) were determined by agar dilution and microbroth dilution assays. Plant extracts were tested for synergistic antimicrobial activity with different antimicrobial agents by checkerboard titration, Etest/agar incorporation assays, and time kill kinetics. Extract treated and untreated bacteria were subjected to transmission electron microscopy to see the effect on bacterial cell morphology. Camellia sinensis extract showed higher antibacterial activity against MDR S. Typhi, alone and in combination with nalidixic acid, than to susceptible isolates.” We further explore anti-staphylococcal activity of *Juglans regia* that lead to the changes in bacterial cell morphology indicating the cell wall of Gram-positive bacteria as possible target of action. The synergistic combination of *Juglans regia* and oxacillin reverted oxacillin resistance of methicillin resistant *Staphylococcus aureus* (MRSA) strains *in vitro*. This study provides novel information about antimicrobial and synergistic activity of *Camellia sinensis* and *Juglans regia* against MDR pathogens

## Introduction

The increase of infections involving multidrug-resistant (MDR) bacteria and of resistance to last resort antimicrobial agents have limited therapeutic options of bacterial infections.

According to the World Health Organization, infectious diseases are the third most significant cause of mortality around the world (http://www.who.int/mediacentre/factsheets/fs310/en/index2.html). The burden of infectious diseases is high in developing countries, as is the emergence of MDR pathogens due to poor health-care facilities, and over-the-counter availability and misuse of antimicrobial agents [1,2]. The frequency of resistance is observed equally among Gram-negative and Gram-positive organisms, although Gram-negative bacteria are prone to develop a MDR phenotype. The high incidence rate of MDR *Pseudomonas* and *Acinetobacter* infections in critically ill patients as well as the presence of MDR *Salmonella* and *Staphylococcus aureus* in normal communities are classic examples of microbiological challenges posed in these geographic locations [3,4,5,6].

In the last fifty years, only two novel classes of antimicrobial compounds such as oxazolidinone and cyclic lipo-peptide have passed clinical trials and are available for clinical use. These agents have undergone analogue development and six drugs were introduced in market in the last decade; however, they are not useful for MDR pathogens due to the rapidly evolving mechanisms of resistance among bacteria [7,8]. Most other compounds do not proceed to clinical trials due to lack of sustained activity and higher toxicity rates. It is clearly understood that progress in the field of drug discovery is far behind what is required to keep up with present day needs. The situation necessitates the development of new antimicrobial agents in rapid fashion.

Plants are historically used to treat infectious diseases. In earlier days people used to discover remedies from the local herbs. People first used plants as food and if results of ingestion were favorable, the plants were linked with some sedative and curative properties. For example, remains of the hollyhock plant, which is still an important herb in phytomedicine, are found in the ancient civilization of the Neanderthals [9]. Scientific evidence supports the hypothesis that several plants are composed of biologically active chemical entities and several drugs in modern day medicine are actually analogues of plant origin substances [10,11,12]. In this study, we aimed to screen the antibacterial activity of *Camellia sinensis* (green tea) and *Juglans regia*, which are commonly used in the Pakistani population. Aqueous and methanolic preparations of plant extracts were tested for antibacterial activity against a wide variety of pathogens alone and in combination with several antimicrobial agents. We further determined their effect on bacterial morphology.

## Materials and Methods

### Bacterial Strains

A total of 350 Gram-positive and Gram-negative strains were obtained from the culture repository of the Immunology and Infectious Diseases Research Laboratory, Department of Microbiology, University of Karachi. Bacterial strains were of clinical and environmental origins (Tables 1 and 2). Clinical strains were isolated from urine, stool, wound, sputum and blood samples, submitted to several pathological laboratories of Karachi for microbiological culture and sensitivity. Environmental isolates were obtained from the culture collection of Department of Microbiology, University of Karachi. Reference strains included *S*. *aureus* ATCC 25923, *Escherichia coli* ATCC 25922, *Salmonella enterica* serovar Typhi ATCC 13311, and *Shigella flexneri* ATCC 9199. Biochemical identification and antimicrobial susceptibility was performed as described previously [13] and antimicrobial susceptibility data were interpreted according to standard breakpoints described by clinical and laboratory standard institute (CLSI) criteria for each antimicrobial agents [14]. The origin and resistance pattern of these bacterial isolates are given in S1 Table. Isolates were stored in Luria-Bertani (LB) broth containing 50% glycerol at -80°C. Log phase bacterial cultures grown in LB broth were used for antibacterial assays.

**Table 1:** Antimicrobial activity of *Camellia sinensis*.

Organisms	Origin	R-type	n	*Camellia sinensis* (green tea)					
				Aqueous			Methanolic		
				Zone (mm)	MIC mg/ml	MBC mg/ml	Zone (mm)	MIC mg/ml	MBC mg/ml
*Staphylococcus aureus*	Clinical	S	40	**17**	0.78	1.56	**20**	0.39	1.56
*Staphylococcus aureus*	Clinical	MRSA	99	**17**	0.19	1.56	**20**	0.39	1.56
*Streptococcus pyogenes*	Clinical	S	05	**17**	0.78	1.56	ND	ND	ND
*Escherichia coli*	Clinical	S	125	07	3.12	5.0	06	5	>5
*Salmonella enterica* serovar Typhi	Clinical	MDR	16	06	1.56	1.56	06	1.25	2.5
*Salmonella enterica* serovar Typhi	Clinical	S	22	06	3.12	3.12	06	2.5	2.5
*Pseudomonas aeuginosa*	Clinical	MDR	02	06	>5	>5	06	>5	>5
*Acinetobacter buamannii*	Clinical	MDR	01	06	>5	>5	06	>5	>5
*Klebseilla pneumoniae*	Clinical	MDR	01	06	3.12	5.0	06	5	>5
*Citrobacter freuendii*	Clinical	MDR	01	06	3.12	5.0	06	5	>5
*Enterobacter cloacae*	Clinical	MDR	01	06	3.12	5.0	06	5	>5
*Bacillus subtilis*	Env.	S	02	**17**	0.78	1.56	**20**	0.78	1.56
*Streptococcus pneumoniae*	Clinical	S	01	**12**	1.56	3.12	**20**	0.78	3.12
*Micrococcus*	Env.	S	01	**13**	0.78	1.56	**20**	0.39	1.56
*Salmonella* Paratyphi A	Clinical	S	08	06	1.56	3.12	06	1.25	2.5
*Shigella species*	Clinical	S	**15**	06	3.12	6.25	ND	ND	ND
*Helicobacter pylori*	Clinical	R A C	02	06	>5	>5	09	2.5	>5
*Helicobacter pylori*	Clinical	S	03	06	>5	>5	09	2.5	>5
*Campylobacter jejuni*	Clinical	S	05	06	>5	>5	06	>5	>5
*S*. *aureus* ATCC 25923,	Reference			20	0.78	1.56	20	0.39	1.56
*Escherichia coli* ATCC 25922,	Reference			06	3.12	5.0	06	5	>5
*Salmonella* Typhi ATCC 13311,	Reference			06	3.12	3.12	06	2.5	2.5
*Shigella flexneri* ATCC 9199	Reference	S		06	>5	>5	06	2.5	>5

S_ pan susceptible; MRSA_ Methicillin resistance *Staphyococcus aureus*; MDR_ Multidrug resistant; MIC_ minimum inhibitory concentration; MBC_ minimum bactericidal concentration; Env._ environmental; R-type_ Resistance phenotype; n_number of isolates; zone_diameter of zone of inhibition; R A C_Rifampicin Ampicillin Chloramphenicol.

**Table 2:** Antimicrobial activity of *Juglans regia*.

				*Juglans regia*					
Organisms	Origin	R-type	N	Aqueous Crude			Methanol		
				Zone mm	MIC mg/ml	MBC mg/ml	Zone mm	MIC mg/ml	MBC mg/ml
*Staphylococcus aureus*	Clinical	MRSA	99	**23**	0.31	0.6	**28**	0.31	1.06
*Staphylococcus aureus*	Clinical	S	40	**>20**	1.25	2.5	**18**	0.53	1.06
*Bacillus subtilis*	Env.	S	01	**20**	0.78	2. 5	**19**	1.06	2.5
Micrococcus	Env.	S	01	**19**	0.78	1.56	**19**	0.78	1.56
*Escherichia coli*	Clinical	S	123	06	>5	> 5	06	1.06	2.1
*Salmonella enterica* serovar Typhi	Clinical	MDR, S	38	**13**	>5	> 5	06	>5	>5
*Pseudomonas aeruginosa*	Clinical	MDR	2	06	>5	> 5	06	>5	>5
*Streptococcus pyogenes*	Clinical	S	05	**15**	1.25	5	**12**	1.25	5
*Streptococcus pneumonia*	Clinical	S	01	06	>5	>5	**12**	1.25	5
*Shigella species*	Clinical	S	15	06	2.5	>5	06	>5	>5
*Enterobacter cloacae*	Clinical	MDR	01	06	5	>5	06	>5	>5
*Pasteurella multocida*.	Clinical	S	02	**15**	1.25	5	09	2.5	5
*Helicobacter pylori*	Clinical	R A C	02	06	>5	>5	09	>5	>5
*Helicobacter pylori*	Clinical	S	03	06	>5	>5	09	>5	>5
*Campylobacter jejuni*	Clinical	S	05	06	>5	>5	06	>5	>5

S_ pan susceptible; MRSA_ Methicillin resistance *Staphyococcus aureus*; MDR_ Multidrug resistant; MIC_ minimum inhibitory concentration; MBC_ minimum bactericidal concentration; Env._ environmental; R A C_ Rifampicin Ampicillin Chloramphenicol; R-type_ Resistance phenotype; n_number of isolates; zone_diameter of zone of inhibition

### Preparation of plant extracts

Plant material included the dried leaves of *Camellia sinensis* (green tea) and the bark of *Juglans regia* (locally referred to as dandasa). The samples were purchased from a local market and identified by Ms. Muneeba Khan, the designated plant taxonomist of the Department of Botany, University of Karachi, Pakistan. Plant material was ground to a powder in a mechanical grinder. Five percent (w/v) aqueous infusion of plant material was prepared in sterile ultrapure laboratory grade water by three successive cycles of heating at 80°C for three minutes. The solutions were filtered using 0.22μm membrane [15]. For methanolic extracts, plant material was soaked into 95% methanol in a ratio of 1: 10 (w/v) for 72 hours at 22°C with vigorous shaking. The extract was subjected to methanol evaporation under vacuumed pressure and residual material was considered as source of methanolic extract. Stock solutions were prepared in DMSO (Merck, Darmstadt, Germany)) and final working volumes were achieved by diluting the stock into Muller Hinton (MH) broth (Oxoid, Hamshire, UK).

### Antimicrobial susceptibility assays

A total of 350 bacterial strains (Tables 1 and 2) were subjected to antimicrobial susceptibility and MIC assays. In addition, reference strains such as *S*. *aureus* ATCC 25923, *Escherichia coli* ATCC 25922, *Salmonella enterica* serovar Typhi ATCC 13311, and *Shigella flexneri* ATCC 9199 were run each time as control for susceptibility and MIC assays. Plant extracts were screened for antimicrobial activity using the agar well diffusion method. Bacterial cultures were inoculated in LB broth for 3 hours at 37°C and turbidity was adjusted in phosphate buffered saline (PBS) to 0.5 McFarland’s index. Using a sterile cotton swab, a bacterial lawn was spread on 90 mm MHA plates containing 6mm wells. Twenty microliters of plant extracts were poured into each well and plates were incubated at 37°C aerobically for 18 hours. Columbia blood agar plates containing 7% lake horse blood (Oxoid, Hamshire, UK) provided a platform for *Helicobacter* and *Campylobacter* based assays. Plates were incubated in microaerophilic conditions using a Campygen kit (Oxoid, Hamshire, UK) at 37°C for 48 hours. The diameter of the zone of bacterial inhibition around each well was measured [16].

Minimum inhibitory concentrations (MICs) were determined by agar dilution and microbroth dilution assays using MH agar and MH broth respectively [17]. The concentrations of the extracts tested ranged from 5000 to 50μg/ml. In the case of agar dilution, experiments were performed on MH agar plates containing various concentrations of extracts (1:20 v/v). Plates were inoculated with ten microliters of the test organism containing 10^5^ CFU log phase bacteria per spot and a total of 20 spots were tested on one plate. In the case of the microbroth dilution assay, sterile flat-bottom 96-well plates were loaded with 100μl of two-fold dilutions of extracts into each well. The starting bacterial inoculum was 1.5x10^5^ CFU/ml. Plates without any plant extracts were served as growth control. The assay plates were incubated as described above. The highest dilution of extract that showed no visible bacterial growth per spot and no turbidity in agar dilution and microbroth dilution assay respectively was considered as MIC. To determine minimum bactericidal concentration (MBC) of extracts, 100μl of MH broth from each well of microbroth assay was sub-cultured on MH agar plates after 24 hours of initial incubation. MH plates were incubated for another 24 hours. The lowest concentration of extract that resulted in no bacterial growth was considered as MBC. Experiments were performed in quadruplicate on five different occasions.

### Synergistic antimicrobial assays

Antimicrobial activity of plant extracts in the presence of different antimicrobial agents, such as oxacillin, tetracycline, nalidixic acid, ofloxacin, chloramphenicol, gentamicin and penicillin was determined. The details about the preprartion of antimicrobial agents are provided in S2 Table. *Camellia sinensis* was tested against representative strains of *Salmonella enterica* serovar Typhi (n = 16) while *Juglans regia* extract was tested against *Staphylococcus aureus* (n = 21). Each combination was first tested by the checkerboard titration method using MH broth in microtiter plates as described previously [18]. Concentrations of extracts ranged from 5000 to 5 μg/ml. The antimicrobial activity of the extract and antibiotic combination was interpreted as one of the following categories: Synergy; indifferent; additive effect; or antagonism. The fractional inhibitory concentration (FIC) of each agent was calculated as the MIC of the agent in combination, divided by the MIC of the agent alone. The interpretation was made on the basis of the fractional inhibitory concentration index (ΣFIC), which is the sum of the FICs of both agents. The FICI results were interpreted as follows: < 0.5 synergy; 0.5 to 1 additive effect; 1–2 indifferent or no effect; and >2 antagonism [19]. Synergistic combinations were further checked using the Etest/agar incorporation assay [20] in which an Etest-strip of each respective antimicrobial agent was placed on MH agar plates containing FIC and 0.5 x FIC of each extract. MH plates containing no antimicrobial agent or extract served as growth control. Plates were incubated at 37°C for 16 hours. Breakpoint values for the resistance of antimicrobial agents were defined per standard criteria.

### Time kill kinetic assay

Time kill kinetic assays were performed on successful synergistic combinations obtained by Etest-strip and checkerboard titration methods. Flasks containing 100 ml MH broth and the drug-plant extract combination were inoculated with a log phase culture of the test organism to a density of 1 x 10^5^ CFU/ml. Test strains included *Salmonella enterica* serovar Typhi (n = 16) and *Staphylococcus aureus* (n = 21). Individual components of each combination, either extract or antibiotic, were added to the control flask to compare the effects of the synergistic combinations to their individual effects on the bacterial growth curve, while no drug was added to the growth control flask. Flasks were incubated for 24 hours at 37°C. Aliquots (100μl) were collected at different time intervals from each flask, serially diluted in PBS, and cultured on MHA plates to obtain colony counts [21]. Curves were constructed by plotting the log_10_ of CFU/ ml versus time. Synergy was defined as ≥2 log_10_ decreases in CFU of organisms treated with the drug combination compared to the most active component of the alone as described previously [22].

### Transmission electron microscopy

On the basis of biological activity, bacterial strains treated with sub-inhibitory concentrations (0.5 x MIC) of *Camellia sinensis* and *Juglans regia* extracts were subjected to transmission electron microscopy to see their effects on bacterial cell morphology as previously described protocol with some modifications [23]. Test organisms (*S*. *aureus* n = 21) were grown in 10ml of MH broth containing sub-inhibitory concentrations (0.5 X MIC) of plant extracts, at 37°C for 18 hours. Broth was centrifuged at 5000 x g and pellets were washed twice with PBS, fixed with gluteraldegyde and OsO_4,_ dehydrated in graded ethanol and resuspended in 500μl PBS. Formvar coated 300-mesh copper grids were coated with 5μl sample suspension for 10 min with the help of sterile tweezers and rinsed once with sterile MilliQ water to remove excessive non-coated material. Copper grids were locally made by the mechanical engineering department of the N. E.D. University of engineering, Karachi. Negative staining was performed by immersing coated grids in 1% uranyl acetate for 20 seconds followed by destaining in sterile MilliQ water and air drying. Grids were observed directly with a JOEL CO-Japan’s JEM 100 transmission electron microscope operating at 80 kV.

## Results

### Antimicrobial activity of *Camellia sinensis* extracts

**Fig 1:**
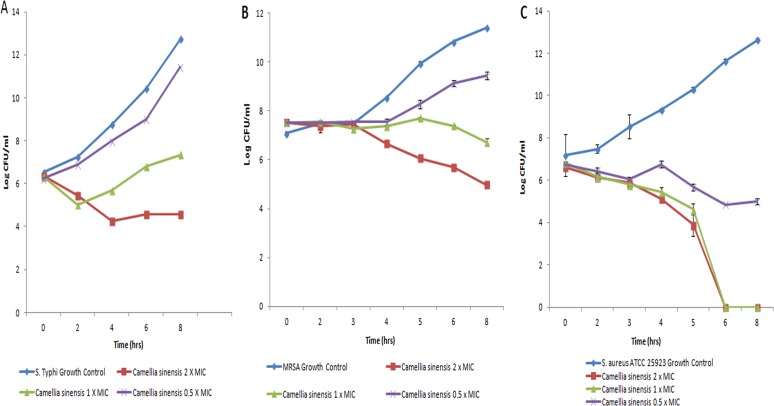
Effect of *Camellia sinensis* on bacterial growth kinetics. Representative bacterial strains of (a) *Salmonella enterica* serovar Typhi, (b) Methicillin resistant *Staphylococcus* aureus (MRSA), and (c) *S*. *aureus* ATCC 25923 (pan—susceptible) were treated with graded concentrations of methanolic extract of *Camellia sinensis* (green tea). Growth cycle of untreated organisms served as growth control. A 2 Log_10_ decrease at any time point from original CFU was considered as significant. Results are presented as an average for three experiments.

Susceptibility studies showed that C*amellia sinensis* (green tea) extracts have higher antimicrobial activity against multidrug resistant *Salmonella* Typhi and sensitive *Salmonella* Paratyphi A than other Gram-negative isolates (Table 1). The *S*. Typhi strains (n = 16) with phenotypes resistant to ampicillin (Amp), tetracycline (T), streptomycin (S), co-trimoxazole (Sxt), nalidixic acid (Na), and ciprofloxacin (Cip) were inhibited by 1.25mg/ml of methanolic and 1.56mg/ml of aqueous extract of green tea. The respective MIC/MBC ratio was 1 and 0.5, and MICs were higher to pan-susceptible strains, however no activity (only 6 mm zone of inhibition) was observed around by agar well diffusion assay. The difference in the data obtained by these assays could be explained by the presence of high molecular weight bioactive compounds which are not readily absorbed and move through solidified agar medium. Time kill kinetics further explained the bacteriostatic effect of green tea extracts on *S*. Typhi (Fig. 1A). No significant antimicrobial activity was observed in other Gram-negative and microaeophilic organisms such as *Pseudomonas aeruginosa*, *Escherichia coli*, *Acinetobacter buamannii*, *Klebseilla pneumoniae*, *Citrobacter freundii* and *shigella species*. Although the MIC of aqueous extract was 3.12 mg/ml, they were not considered active due to higher MIC values in methanolic extract and no activity by agar well diffusion assay. Green tea was found to be very active against Gram-positive organisms, including *Staphylococcus aureus*, *Microccocus*, *Streptococcus pneumonia* and *Bacillus subtilis*. The methanolic extract inhibited the growth of *S*. *aureus* with a 20mm zone of inhibition and an average MIC of 0.39 mg/ml and MBC 1.56 mg/ml. Although insignificant, MICs of aqueous extracts were lower for methicillin-resistant *Staphylococcus aureus* (MRSA) compared to sensitive reference and clinical strains of *S*. *aureus* (Table 1 and S3 Table). Dose dependent cidal effect was observed against drug-resistant and sensitive strains on 24 and 6 hours respectively (Fig. 1B and 1C).

### Antimicrobial activity of *Juglans regia* extracts

**Fig 2:**
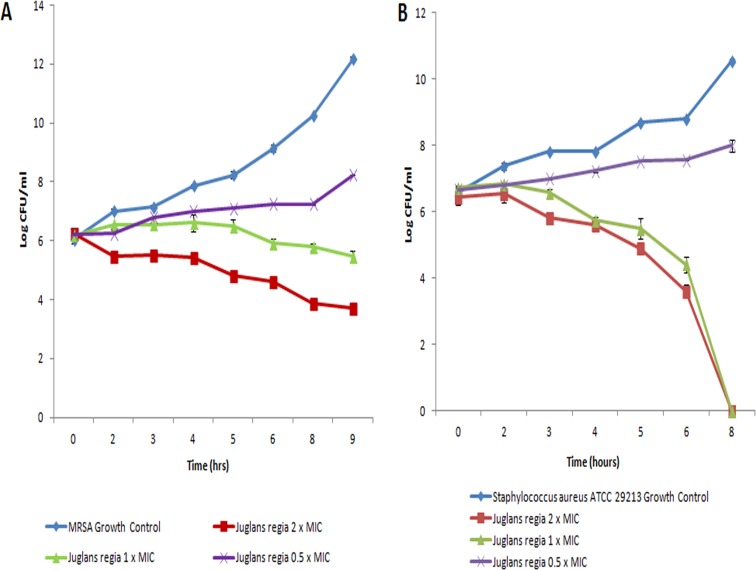
Effect of *Juglans regia* on bacterial growth kinetics. (a) Methicillin resistamt *Staphylococcus* aureus (MRSA) and (b) *S*. *aureus* ATCC 25923 (pan—susceptible) were treated with graded concentrations of aqueous extract of *Juglans regia* (Dandasa). Growth cycle of untreated organisms served as growth control. A 2Log_10_ decrease at any time point from original CFU was considered as significant. Results are presented as an average for three experiments.

The most potent inhibitory effect of *Juglans regia* extracts was observed on MRSA strains with MICs 0.31 mg/ml and a zone of inhibition of more than 20 mm around aqueous and methanolic extracts (Table 2). Interestingly, two- to four-fold increases in MIC were found when drug-sensitive clinical strains of *S*. *aureus* were tested, but the MIC/MBC ratio was 0.5 in both cases. In contrast with green tea, *Juglans regia* did not show activity with Gram-negative bacteria; only *E*. *coli* was found to be susceptible at 1.06 mg/ml of methanolic extract with no zone of inhibition. Time kill kinetics showed a dose dependent bactericidal effect on methicillin-resistant and sensitive *S*. *aureus* strains, indicating strong anti-staphylococcal activity of *Juglans regia* irrespective of the spectrum of resistance against other antimicrobial agents (Fig. 2). Our data clearly indicate strong antimicrobial activity of *Juglans regia* against Gram-positive organisms, particularly *Staphylococcus* species.

### Synergistic activity of plant extracts with antimicrobial agents

**Fig 3:**
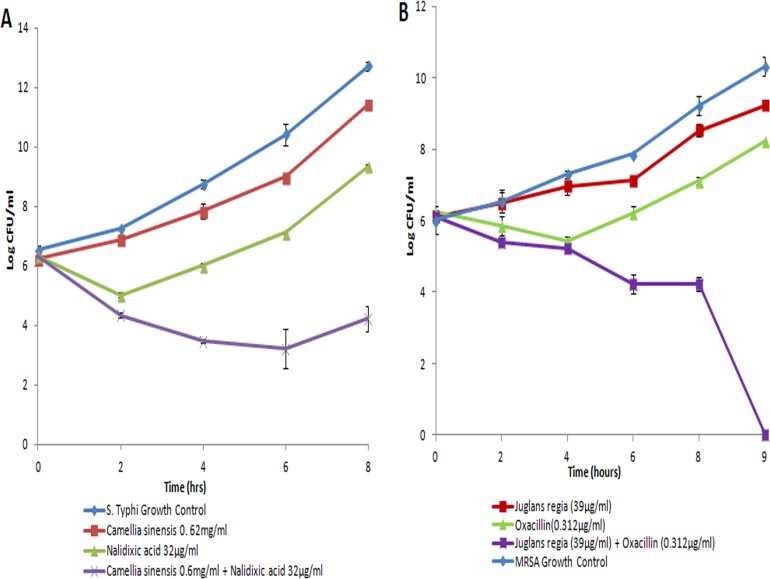
Effect of synergistic antimicrobial combinations on growth kinetics of MDR pathogens. Synergistic antimicrobial combinations of (a) *Camellia sinensis* and nalidixic acid and (b) *Juglans regia* and oxacillin were tested for antimicrobial effect on growth kinetics of MDR *Salmonella enterica* serovar Typhi and Methicillin Resistant *Staphylococcus aureus* respectively. Organisms were grown in MH broth in the presence of plants extracts alone and in combination with antibiotics and samples were taken for colony counts at different time intervals. Control organisms were grown in MH broth only. A 2Log_10_ decrease at any time point from original CFU was considered as significant. Results are given as average for three experiments.

A number of antibiotic combinations were checked with synergistic activity of *Camellia sinensis* and *Juglans regia* extracts and two different combinations showed strong synergistic activity (Table 3). When *Camellia sinensis* was combined with nalidixic acid, it was able to inhibit *S*. Typhi at sub-MIC levels. We also observed a significant reduction in MICs of nalidixic acid, which explains the strong synergy (FICI 0.37) in this combination (Table 3). However, the MICs of nalidixic acid did not reach breakpoint level (8μg/ml) for the MDR strain (R type-Amp C Sxt T Na). Time kill kinetics showed synergy at 2, 4, 6 and 8 hours (Fig. 3A).

**Table 3:** Synergistic antimicrobial activity of plant extracts with different antimicrobial agents.

Bacteria	R-type		Antimicrobial Agent	MIC of Antimicrobial Agent (μg/ ml)		MIC of *Camellia sinensis* (mg/ml)		FICI	Outcome
		n		Alone	combination	alone	combination		
*Salmonella enterica* serovar Typhi	AmpCSxtTNa	3	Chloramphenicol	512	256	2.5	2.5	1.5	Indifferent
			Tetracyclin	1	0.5	2.5	2.5	1.5	Indifferent
			Nalidixic acid	256	32	2.5	0.62	0.37	Synergistic
	Amp C Sxt T	7	Nalidixic acid	8	1	2.5	0.62	0.37	Synergistic
	Pan-susceptible	6	Nalidixic acid	0.5	0.12	2.5	1.25	0.74	Additive
			Tetracyclin	0.25	0.25	2.5	1.25	1.5	Indifferent
			Chloramphenicol	2	2	2.5	0.75	1.3	Indifferent
						**MIC of *Juglans regia*** (mg/ml)			
*Staphylococcus aureus*	MRSA	15	Gentamicin	32	16	0.31	0.31	1.5	Indifferent
			Penicillin	4	1	0.31	0.15	0.7	Additive
			Ofloxacin	16	16	0.31	0.31	2	Indifferent
			Oxacillin	≥20	0.312	0.31	0.039	0.14	Synergistic
			Tetracycline	64	64	0.31	0.96	4	Antagonism
	Pan-susceptible	6	Gentamicin	2	2	1.25	1.25	2	Indifferent
			Penicillin	0.05	0.02	1.25	0.65	0.92	Additive
			Ofloxacin	0.25	0.25	1.25	1.25	2	Indifferent
			Oxacillin	0.5	0.312	1.25	0.37	0.92	Additive
			Tetracycline	1	1	1.25	2.5	3	Antagonism

FICI_ fractional inhibitory concentration index; R-type_ Resistance phenotype; n_number of isolates; zone_diameter of zone of inhibition; MIC_ minimum inhibitory concentration

In the case of *Juglans regia*, 10 times reduction in MICs was observed against *Staphylococcus aureus* when used in combination with oxacillin. In this combination, oxacillin was able to inhibit MRSA strains at 0.312μg/ml, which was 64 times lower than the MIC of oxacillin alone and indicates a reversion of methicillin resistance if used along with *Juglans regia*. In time kill kinetics, the *Juglans regia* and oxacillin combination exhibited synergy at 3, 4, 5, 8 and 9 hours in 10/10 MRSA strains (Fig. 3B). A complete cidal effect was observed in 9 hours, which persisted to 24 hours of incubation.

### Effect on bacterial cell morphology

**Fig 4:**
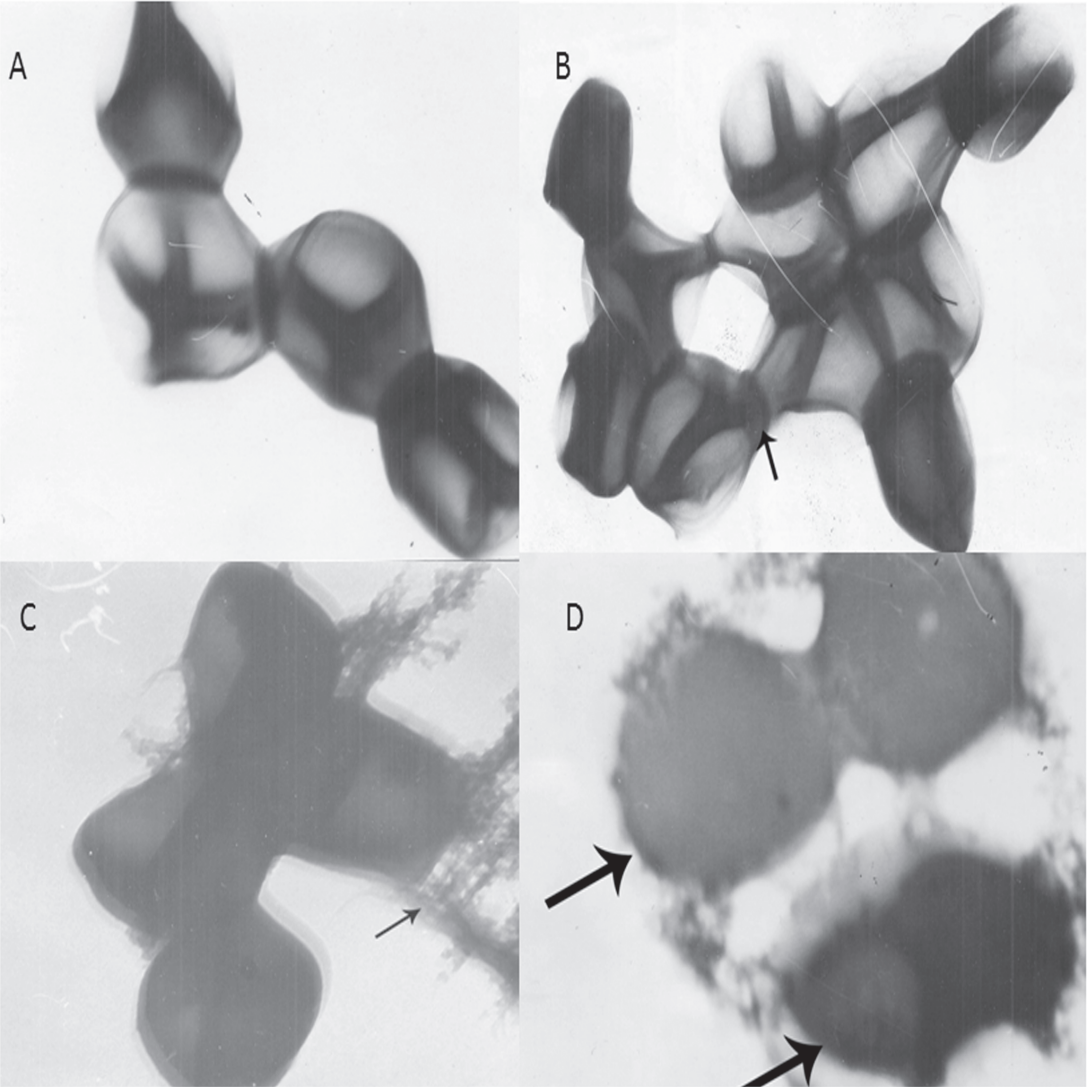
Effect of *Juglans regia* on cell morphology of MRSA. Change in cellular morphology of MRSA was observed by Transmission Electron Microscopy, A: Control cell, B-D shows bacterial cells treated with *Juglans regia*. B: Intracellular thick material. C: covering on unknown material on cell surface. D: Hollow and de-shaped bacteria.

Transmission electron microscopy showed characteristic morphological changes in MRSA after receiving treatment with *Juglans regia* extract for 18 hours. In contrast with untreated growth control (Fig. 4A), thick intracellular masses were observed in bacterial cells treated with *Juglans regia* (Fig. 4B) that further led to the deformation of bacterial cells (Fig. 4C and 4D); however, we did not find complete deformation of the cell. We also observed that *Juglans regia-*treated deformed bacteria were coated with thread-like material, indicating the presence of extract on the cellular surface (Fig. 4). Prominent changes in bacterial cell morphology indicate that bacterial cell membrane permeability and viscosity was compromised by the *Juglans regia* extract.

## Discussion

*Camellia sinensis* belongs to the family Theaceae of tea plants. It is widely cultivated in tropical and subtropical regions all over the world. Previous studies reported the antimicrobial activity of green tea leaves to Gram-positive organisms [24,25,26]; however, discrepancies were found regarding its activity in relation to Gram-negative rods [27,28,29]. In this study, we observed that *Camellia sinensis* showed higher antibacterial activity against MDR *S*. Typhi than to other Gram-negative isolates. By testing a variety of clinical strains in a time kill kinetic assay, we further showed that green tea extract is bacteriostatic in nature and more efficient against MDR strains of *S*. Typhi compared to pan-susceptible strains. Previous studies have reported that tea catechins are bioactive components present in green tea particularly they are active against Gram positive bacteria[30,31], however none of them is seen to be antimicrobial against Gram negative bacteria. To the best of our knowledge this is the first report providing strong evidence of the antimicrobial activity of green tea against drug-resistant Gram-negative bacteria.

*Juglans regia* (walnut tree) is a common temperate forest tree found in Asia. The dried bark of this treeis traditionally used in Pakistan to improve oral hygiene and promote teeth brightening. The tree is rich in polyphenols and in previous studies the antimicrobial activity of this plant was reported against bacteria and fungi [32,33,34]; however, these studies were focused on the tree’s leaves instead of the bark. In this study, we tested *Juglans regia* bark extracts against a wide range of bacterial pathogens and susceptibility data showed that the extract is selectively active against Gram-positive bacteria. We observed strong anti-staphylococcal activity of *Juglans regia* that led to the changes in bacterial cell morphology. The study suggests that *Juglans regia* might affect how metabolic processes occur in bacterial cell walls and cell membranes. In contrast with the findings of Nariman et al. [35], no activity was observed against microaerophilic organisms such as *Helicobacter* and *Campylobacter*. The observations warrant further investigation for the search of compound(s) present in *Juglans regia* bark that leads to the development of novel antimicrobial agent.

In this study, we investigated antimicrobial activity of plant extracts by several methods including agar well diffusion, agar dilution and microbroth dilution assays. Significant differences were not observed in the MICs of *Camellia sinensis* and *Juglans regia* extracts obtained by agar dilution and microbroth dilution methods. However the growth of certain strains was not inhibited by agar well diffusion assay. Given that the assay was performed with crude extract, we hypothesize that presence of high molecular weight components might hinder in the uniform distribution and movement of bioactive compound through solidified agar medium.

Although the antimicrobial activity of these plants has been previously reported, little is known about their interaction with antimicrobial agents which are commonly prescribed to treat MDR bacterial infections. Here we tested the synergistic activity of *Camellia sinensis* and *Juglans regia* extracts with a wide range of antimicrobial agents. *Camellia sinensis* showed synergistic activity with nalidixic acid (FICI 0.37) against MDR *S*. Typhi strains. Since *Camellia sinensis* is bacteriostatic for Gram-negative rods, we hypothesize that this combination might prevent the recurrence of bacterial growth caused by antibiotic selective mutants towards the end of the disease’s course; therefore, it is worthwhile to test *Camellia sinensis* and its compounds for synergistic activity with quinolones *in vivo*.

It was very surprising to see that *Juglans regia* was able to reverse oxacillin resistance in MRSA. Oxacillin is a β-lactam antibiotic that inhibits cell wall peptidoglycan through binding and competitive inhibition of penicillin binding proteins (PBPs) [36]. Activation of *mecA* gene and gene variants led to the formation of PBP2a, which binds with β-lactam antibiotics in lower affinity, resulting in drug resistance[37,38]. Therefore, unavailability of PBP2a might resume antimicrobial action of oxacillin in MRSA strains. We have already shown that *Juglans regia* alone induces ultrastructure changes in MRSA, indicating significant changes in the molecular machinery of bacterial cells. We speculate that the addition of *Juglans regia* might interfere in the synthesis or transportation of PBP2a levels on the cell membrane. Previous studies also showed similar effects on oxacillin resistance by different compounds such as phenothiazine, chloropromazine, thioridazine, inducing physiological damage to bacterial cell membranes [39,40]. Further studies are warranted to understand the exact mechanism of action. This study is the first to report the synergistic antimicrobial effects of *Juglans regia* in combination with β-lactam antibiotics leading to the reversion of oxacillin resistance.

In a separate study, we tested these extracts for toxic effects on human RBCs. *Camellia sinensis* was found to be non-toxic on the concentrations tested for antimicrobial activity while *Juglans regia* showed toxicity at concentrations 100 times higher than the one showed antimicrobial activity (data not shown). The significant increase in toxic concentrations for human RBCs demonstrates the feasibility of *Jeglans regia* to test for further bioactive compound purification activities.

This study provides novel information about the antimicrobial potential of *Camellia sinensis* and *Juglans regia* to MDR pathogens. Further studies investigating the mechanism of synergistic action are important to prove candidacy of these substances for antimicrobial therapy.

## Supporting Information

S1 Table Antimicrobial resistance pattern of bacterial isolates used in this study. (XLS)

S2 Table Detailed information about antimicrobial agents. (XLS)

S3 Table Raw data for antimicrobial activities of Camellia sinensis and Juglans regia against 350 bacterial isolates. (XLS)

## References

[1] Vincent JL, Rello J, Marshall J, Silva E, Anzueto A, Martin CD, et al International study of the prevalence and outcomes of infection in intensive care units. JAMA. 2009; 302: 2323–2329. 10.1001/jama.2009.1754 19952319

[2] Allegranzi B, Nejad SB, Combescure C, Graafmans W, Attar H, Donaldson L, et al Burden of endemic health-care-associated infection in developing countries: systematic review and meta-analysis. Lancet 2011;377: 228–241. 10.1016/S0140-6736(10)61458-4 21146207

[3] Akram F, Pietroni MA, Bardhan PK, Bibi S, Chisti MJ. Prevalence, clinical features, and outcome of *Pseudomonas* bacteremia in under-five diarrheal children in Bangladesh. ISRN Microb 2014;469758 10.1155/2014/469758 24734204 PMC3966484

[4] Vernet G, Mary C, Altmann DM, Doumbo O, Morpeth S, Bhutta ZA, et al Surveillance for Antimicrobial Drug Resistance in Under-Resourced Countries. Emerg Infect Dis. 2014; 20: 434–441. 10.3201/EID2003.121157 24564906 PMC3944851

[5] Iyer A, Kumosani T, Azhar E, Barbour E, Harakeh S. High incidence rate of methicillin-resistant *Staphylococcus aureus* (MRSA) among healthcare workers in Saudi Arabia. J Infect Dev Ctries. 2014; 8: 372–378. 10.3855/jidc.3589 24619270

[6] Jain S, Chugh TD. Antimicrobial resistance among blood culture isolates of *Salmonella enterica* in New Delhi. J Infect Dev Ctries. 2013;7: 788–795. 10.3855/jidc.3030 24240035

[7] Coates AR, Halls G, Hu Y. Novel classes of antibiotics or more of the same? Br J Pharm. 2011;163: 184–194. 10.1111/j.1476-5381.2011.01250.x 21323894 PMC3085877

[8] Moellering RC Jr. MRSA: the first half century. J Antimicrob Chemother. 2012;67: 4–11. 10.1093/jac/dkr437 22010206

[9] Cowan MM. Plant products as antimicrobial agents. Clin Microb Rev. 1999;12: 564–582. 10515903 10.1128/cmr.12.4.564 PMC88925

[10] Ghosh S, Chisti Y, Banerjee UC. Production of shikimic acid. Biotech Advances 2012;30: 1425–1431. 10.1016/j.biotechadv.2012.03.001 22445787

[11] Begum S, Naqvi SQZ, Ahmed A, Tauseef S, Siddiqui BS. Antimycobacterial and antioxidant activities of reserpine and its derivatives. Nat Prod Res. 2012;26: 2084–2088. 10.1080/14786419.2011.625502 22273392

[12] Abreu AC, McBain AJ, Simoes M. Plants as sources of new antimicrobials and resistance-modifying agents. Nat Prod Rep. 2012;29: 1007–1021. 10.1039/c2np20035j 22786554

[13] Ali NH, Farooqui A, Khan A, Khan AY, Kazmi SU. Microbial contamination of raw meat and its environment in retail shops in Karachi, Pakistan. J Infect Dev Ctries. 2010; 4:382–388. 20601790

[14] Cockerill FR, Patel JB, Alder J, Bradford PA, Dudley MN, et al. Performance standards for antimicrobial susceptibility testing; twenty third informational supplement: Clinical and Laboratory Standards Institute (CLSI). 2013.

[15] Ali NH, Kazmi SU, Faizi S. Activity of synergistic combination Amoxy-cassia against Salmonella. Pak J Pharm Sci. 2007;20: 140–145. 17416570

[16] Rojas JJ, Ochoa VJ, Ocampo SA, Muñoz JF. Screening for antimicrobial activity of ten medicinal plants used in Colombian folkloric medicine: A possible alternative in the treatment of non-nosocomial infections. BMC Complement Altern Med. 2006;6: 2 16483385 10.1186/1472-6882-6-2 PMC1395329

[17] Klančnik A, Piskernik S, Jeršek B, Možina SS. Evaluation of diffusion and dilution methods to determine the antibacterial activity of plant extracts. J Microb Met. 2010;81: 121–126. 10.1016/j.mimet.2010.02.004 20171250

[18] Timurkaynak F, Can F, Azap ÖK, Demirbilek M, Arslan H, Karaman SO. In vitro activities of non-traditional antimicrobials alone or in combination against multidrug-resistant strains of *Pseudomonas aeruginosa* and *Acinetobacter baumannii* isolated from intensive care units. Int J Antimicrob Ag. 2006;27: 224–228. 16464562 10.1016/j.ijantimicag.2005.10.012

[19] Ahmad A, van Vuuren S, Viljoen A. Unravelling the complex antimicrobial interactions of essential oils—the case of *Thymus vulgaris* (Thyme). Molecules 2014;19: 2896–2910. 10.3390/molecules19032896 24662066 PMC6271043

[20] Cetin ES, Tekeli A, Ozseven AG, Us E, Aridogan BC. Determination of in vitro activities of polymyxin B and rifampin in combination with ampicillin/sulbactam or cefoperazone/sulbactam against multidrug-resistant *Acinetobacter baumannii* by the E-test and checkerboard methods. Jap J Infect Dis. 2013;66: 463–468. 24270131 10.7883/yoken.66.463

[21] Petersen PJ, Labthavikul P, Jones CH, Bradford PA. In vitro antibacterial activities of tigecycline in combination with other antimicrobial agents determined by chequerboard and time-kill kinetic analysis. J Antimicrob Chemother 2006;57: 573–576. 16431863 10.1093/jac/dki477

[22] Sopirala MM, Mangino JE, Gebreyes WA, Biller B, Bannerman T, Balada-Llasat JM. Synergy testing by Etest, microdilution checkerboard, and time-kill methods for pan-drug-resistant *Acinetobacter baumannii* . Antimicrob Ag Chemother. 2010;54: 4678–4683. 10.1128/AAC.00497-10 20713678 PMC2976112

[23] Hartmann M, Berditsch M, Hawecker J, Ardakani MF, Gerthsen D, Ulrich AS. Damage of the bacterial cell envelope by antimicrobial peptides gramicidin S and PGLa as revealed by transmission and scanning electron microscopy. Antimicrob Ag Chemother. 2010;54: 3132–3142. 10.1128/AAC.00124-10 20530225 PMC2916356

[24] Hamilton-Miller J. Antimicrobial properties of tea (*Camellia sinensis* L.). Antimicrob Ag Chemother 1995;39: 2375–2377. 8585711 10.1128/aac.39.11.2375 PMC162950

[25] Chan EW, Soh EY, Tie PP, Law YP. Antioxidant and antibacterial properties of green, black, and herbal teas of *Camellia sinensis* . Pharmacognosy Res. 2011;3: 266–272. 10.4103/0974-8490.89748 22224051 PMC3249787

[26] Hamilton-Miller J, Shah S. Activity of the tea component epicatechin gallate and analogues against methicillin-resistant *Staphylococcus aureus* . J Antimicrob Chemother. 2000;46: 852–853. 11062217 10.1093/jac/46.5.852

[27] Shetty M, Subbannayya K, Shivananda P. Antibacterial activity of tea (*Camellia sinensis*) and coffee (*Coffee arabica*) with special reference to *Salmonella* Typhimurium. J Comm Dis. 1994;26: 147–150. 7868837

[28] Taguri T, Tanaka T, Kouno I. Antimicrobial activity of 10 different plant polyphenols against bacteria causing food-borne disease. Biol Pharm Bull. 2004;27: 1965–1969. 15577214 10.1248/bpb.27.1965

[29] Gordon NC, Wareham DW. Antimicrobial activity of the green tea polyphenol (−)-epigallocatechin-3-gallate (EGCG) against clinical isolates of *Stenotrophomonas maltophilia* . Int J Antimicrob Ag. 2010;36: 129–131. 10.1016/j.ijantimicag.2010.03.025 20472404

[30] Yam T, Shah S, Hamilton‐Miller J. Microbiological activity of whole and fractionated crude extracts of tea (*Camellia sinensis*), and of tea components. FEMS Microb Lett. 1997;152: 169–174. 9228784 10.1111/j.1574-6968.1997.tb10424.x

[31] Zhao W-H, Hu Z-Q, Okubo S, Hara Y, Shimamura T. Mechanism of synergy between epigallocatechin gallate and β-lactams against methicillin-resistant *Staphylococcus aureus* . Antimicrob Ag Chemother. 2001;45: 1737–1742. 11353619 10.1128/AAC.45.6.1737-1742.2001 PMC90539

[32] Pereira JA, Oliveira I, Sousa A, Valentão P, Andrade PB, Ferreira IC. Walnut (*Juglans regia* L.) leaves: Phenolic compounds, antibacterial activity and antioxidant potential of different cultivars. Food Chem Toxicol. 2007;45: 2287–2295. 17637491 10.1016/j.fct.2007.06.004

[33] Oliveira I, Sousa A, Ferreira IC, Bento A, Estevinho L, Pereira JA. Total phenols, antioxidant potential and antimicrobial activity of walnut (*Juglans regia* L.) green husks. Food Chem Toxicol. 2008;46: 2326–2331. 10.1016/j.fct.2008.03.017 18448225

[34] Noumi E, Snoussi M, Hajlaoui H, Valentin E, Bakhrouf A. Antifungal properties of *Salvadora persica* and *Juglans regia* L. extracts against oral *Candida* strains. Eu J Clin Microbiol Infect Dis. 2010;29: 81–88. 10.1007/s10096-009-0824-3 19899011

[35] Nariman F, Eftekhar F, Habibi Z, Falsafi T. Anti‐*Helicobacter pylori* activities of six Iranian plants. Helicobacter 2004;9: 146–151. 15068416 10.1111/j.1083-4389.2004.00211.x

[36] Grubb W. Genetics of MRSA. Rev Med Microbiol. 1998;9: 153–162.

[37] Pinho MG, Filipe SR, de Lencastre Hn, Tomasz A. Complementation of the essential peptidoglycan transpeptidase function of penicillin-binding protein 2 (PBP2) by the drug resistance protein PBP2A in *Staphylococcus aureus* . J Bacteriol. 2001;183: 6525–6531. 11673420 10.1128/JB.183.22.6525-6531.2001 PMC95481

[38] Beck WD, Berger-Bächi B, Kayser FH. Additional DNA in methicillin-resistant *Staphylococcus aureus* and molecular cloning of mec-specific DNA. J Bacteriol. 1986;165: 373–378. 3003024 10.1128/jb.165.2.373-378.1986 PMC214427

[39] Klitgaard JK, Skov MN, Kallipolitis BH, Kolmos HJ. Reversal of methicillin resistance in *Staphylococcus aureus* by thioridazine. J Antimicrob Chemother 2008;62: 1215–1221. 10.1093/jac/dkn417 18836185

[40] Kristiansen JE, Hendricks O, Delvin T, Butterworth TS, Aagaard L, Christensen JB. Reversal of resistance in microorganisms by help of non-antibiotics. J Antimicrob Chemother. 2007;59: 1271–1279. 17403708 10.1093/jac/dkm071

